# The corneo-scleral junction assessed with optical coherence tomography

**DOI:** 10.1371/journal.pone.0278884

**Published:** 2022-12-09

**Authors:** Maria Muzyka-Woźniak, Adam Oleszko, Łukasz Stróżecki, Sławomir Woźniak

**Affiliations:** 1 Ophthalmology Clinical Centre SPEKTRUM, Wrocław, Poland; 2 Department of Anaesthesiology and Intensive Therapy, Wroclaw Medical University, Wrocław, Poland; 3 Department of Human Morphology and Embriology, Department of Anatomy, Wroclaw Medical University, Wrocław, Poland; Saarland University, GERMANY

## Abstract

**Purpose:**

To evaluate corneo-scleral junction (CSJ) using anterior segment optical coherence tomography (AS-OCT) and describe the pattern of cornea and sclera interfusion based on tissue reflectivity.

**Methods:**

This prospective observational study enrolled candidates for vision correction. Eyes with previous ocular surgery or irregular corneas were excluded. Temporal and nasal CSJ width and reflectivity patterns were assessed with AS-OCT horizontal scans. Correlations between manual and automated variables and multivariate linear regression analyses with age and spherical equivalent were performed.

**Results:**

101 right eyes were analysed. Temporal CSJ was wider (median 1.62; 1.13 to 2.22 mm) compared to the nasal side (median 1.18; 0.73 to 1.80 mm) (*p*<.0001). The temporal CSJ width showed negative correlation with ipsilateral anterior chamber angle measurements and positive correlation with horizontal visible iris diameter (HVID). These relationships were not statistically significant for the nasal CSJ width. No significant correlations with age or refractive error were observed at both sides. The pattern of temporal CSJ reflectivity was mostly V- or U-shaped. The eyes with V-shaped temporal CSJ had significantly larger HVID than the eyes with irregular temporal CSJ. The nasal CSJ presented irregular reflectivity in 47% of cases.

**Conclusions:**

The temporal CSJ was wider and had regular (V or U-shaped) reflectivity patterns, while nasal CSJ was narrower and more irregular. The CSJ width was independent of age and refractive error and could not be predicted from other parameters. The HVID measurement accuracy may benefit from CSJ analysis based on AS-OCT.

## Introduction

The corneo-scleral junction (CSJ) or the corneal limbus is a complex, 3-dimensional and circumferential structure. The surgical limbus is referred to the en-face view of the eye and transparency of the anatomical structures. The point of transition between transparent and non transparent tissue at the horizontal corneal meridian serves as the basis for the “white-to white” (WTW) distance estimation. The exact determination is difficult, because there is no definite anatomical border between cornea and sclera. Histologically, the fibers of corneal and scleral collagen are intertwined.

WTW is a crucial measurement for the posterior chamber phakic intraocular lens sizing. It is also used as one of the variables in many formulas used for intraocular lens (IOL) power calculation. WTW, also labelled as horizontal visible iris diameter (HVID), is automatically acquired from the frontal eye image by most of the modern anterior segment imaging devices: optical biometers, optical coherence tomographers (OCT), Scheimpflug cameras and topographers. However, differences between devices are reported in many studies, and the measurements are not interchangeable [1, 2]. The main cause for that may be the different image acquisition and processing. Furthermore, the results may be influenced by CSJ width and shape [3]. The complexity of the transverse section of the CSJ can be clearly visualised with anterior segment OCT (AS-OCT).

The aim of our study was to measure the average CSJ dimensions using AS-OCT images and describe the specific reflectivity pattern of interfusion between corneal and scleral tissue. To the best of our knowledge, an anatomical description of the corneal limbus based on AS-OCT has not been published so far.

## Material and methods

### Study design

This prospective study involved consecutive healthy adult white Caucasian patients who presented for laser vision correction at a private hospital (Ophthalmology Clinical Centre SPEKTRUM, Wroclaw, Poland) from February 2021 to August 2021. The study was approved by the Wroclaw Medical University ethics committee (No. 77/2021) prior to initiation and followed the tenets of the Declaration of Helsinki. Subjects gave written informed consent after explanation of study procedures.

The criteria for exclusion were previous intraocular or corneal surgery, irregular cornea, severe dry eye disease, glaucoma and cataract. Contact lens users had to refrain from wearing lenses at least 3 days before the examination. All subjects underwent a complete ophthalmic examination which included visual acuity, refraction and slit-lamp biomicroscopy. The cycloplegic subjective refraction and fundoscopy were assessed after image acquisition.

Anterior segment imaging was performed on MS-39 (CSO Italy) with PHOENIX software version 3.7.0108. The MS-39 combines Placido disk corneal topography with high resolution spectral domain OCT-based anterior segment tomography. The OCT source has a wavelength of 845 nm, image field 16 mm x 8 mm and axial resolution in tissue > 3.6 μm and transversal resolution of 35 μm (in air). The measurement accuracy is class A according to UNI EN ISO 19980–2012. The repeatability of MS-39 anterior segment measurements has been reported previously [4].

All exams were performed on normal pupil, without any pharmacological pupil dilation, in the same dim ambient lighting conditions (0.02 Lux). During the image acquisition, subjects were asked to refrain from blinking and to fixate on the instrument’s internal fixation target. Each patient had three consecutive exams, and the exam with the best quality was selected for analysis. The acquisition quality check included fixation, sections coverage and keratoscopy coverage and centration. Eyes labelled as keratoconus or keratoconus suspect (as assessed with PHOENIX software) were excluded.

#### AS-OCT automated measurements

MS-39 automated measurements were recorded: scleral spur to scleral spur (SS-SS), HVID (adjusted by the PHOENIX software version 3.7.01.08), central corneal thickness (CCT), temporal peripheral corneal thickness (CTt) and nasal peripheral corneal thickness (CTn) as measured on pachymetry map, anterior corneal curvature radius at flat meridian (K1ant) and steep meridian (K2ant), posterior corneal curvature radius at flat meridian (K1post) and steep meridian (K2post) and aqueous depth (AD). All curvature radiuses were measured at 7 mm zone. Additionally, following angle parameters were measured: angle opening distance (AOD 500 and AOD 750) and trabecular space area (TISA 500 and TISA 750). AOD was defined as a perpendicular distance between a point 500 μm (AOD 500) or 750 μm (AOD 750) anterior to the scleral spur and the opposing iris. TISA was defined as a trapezoidal area (TISA 500 or 750) bounded by the AOD (500 or 750, respectively), the anterior iris surface, the inner corneo-scleral wall and the perpendicular distance between the scleral spur and the opposing iris.

#### AS-OCT manual measurements

**Fig 1:**
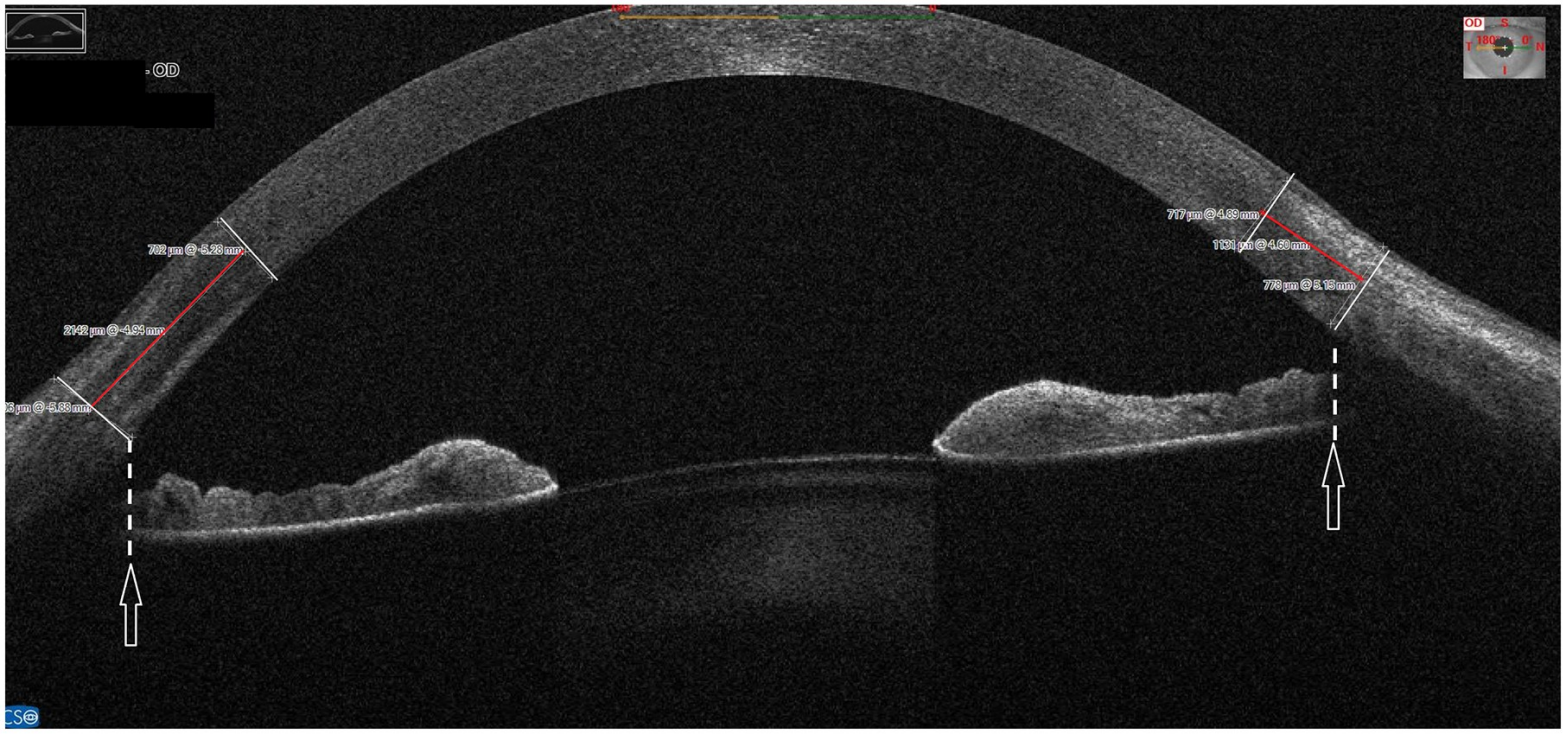
The assessment of corneo-scleral junction width. The horizontal AS-OCT scan of the right eye with callipers set to measure the corneo-scleral junction width (red lines) as a distance between last optically clear corneal section and first optically opaque scleral section. Determination of posterior limbal border was supported with the optical shadowing of iris from the scleral tissue (white arrows). Dashed lines perpendicular to the iris, with a starting point at the last visible iris pigment epithelium, point at the first fully opaque scleral section.

All CSJ width measurements were fully manual, performed on MS-39 single images along the horizontal meridian. The MS-39 built-in callipers with cartesian coordinate system were used for each eye to assess the CSJ width (Fig 1). It was defined as a distance between the last optically clear corneal section and the first optically opaque scleral section. Two transverse lines were drawn: the first–to separate the transition zone from the fully clear (low reflective) cornea; the second–to separate the transition zone from the fully opaque (highly reflective) sclera. The separation was based on tissue reflectivity, which is homogeneous in cornea and sclera and non-homogeneous in the transition zone. Each line, perpendicular to the outer ocular surface, was drawn 2 times, and when their position differed by less than 0.05 mm, the drawing was saved. If the difference was larger, then the whole process was repeated. The value 0.05 mm was based on measurements of the first 10 eyes performed by two independent examiners (MMW, SW). Finally, the distance between two lines was measured with a calliper placed in the middle of corneal thickness, at right angles to the limiting lines. The measurements were done separately for the temporal (CSJt) and nasal side (CSJn) on the 180° horizontal scan with 200% magnification. Determination of the inner posterior limbal border was supported by optical shadowing of iris from scleral tissue: a straight line perpendicular to iris, with a starting point at the last visible iris pigment epithelium, points at the first fully opaque scleral section. All measurements were performed by the same experienced examiner (MMW). The ease of finding and determining the position of separating lines was noted accordingly to the following scale: 1—very distinct, easy determination, 2 –moderate ease, 3 –indistinct separation, difficult to determine. The difference between CSJt and CSJn was calculated as CSJ delta (CSJΔ).

For intra-observer repeatability three independent manual CSJt and CSJn width measurements were conducted by investigator 1 (MMW) using 25 randomly assigned images. For inter-observer reproducibility the same steps were repeated by investigator 2 (SW), who was blinded to investigator 1 findings. The observers had different levels of experience in working with OCT images, one being a relative novice (SW) and the other being more experienced (MMW).

**Fig 2:**
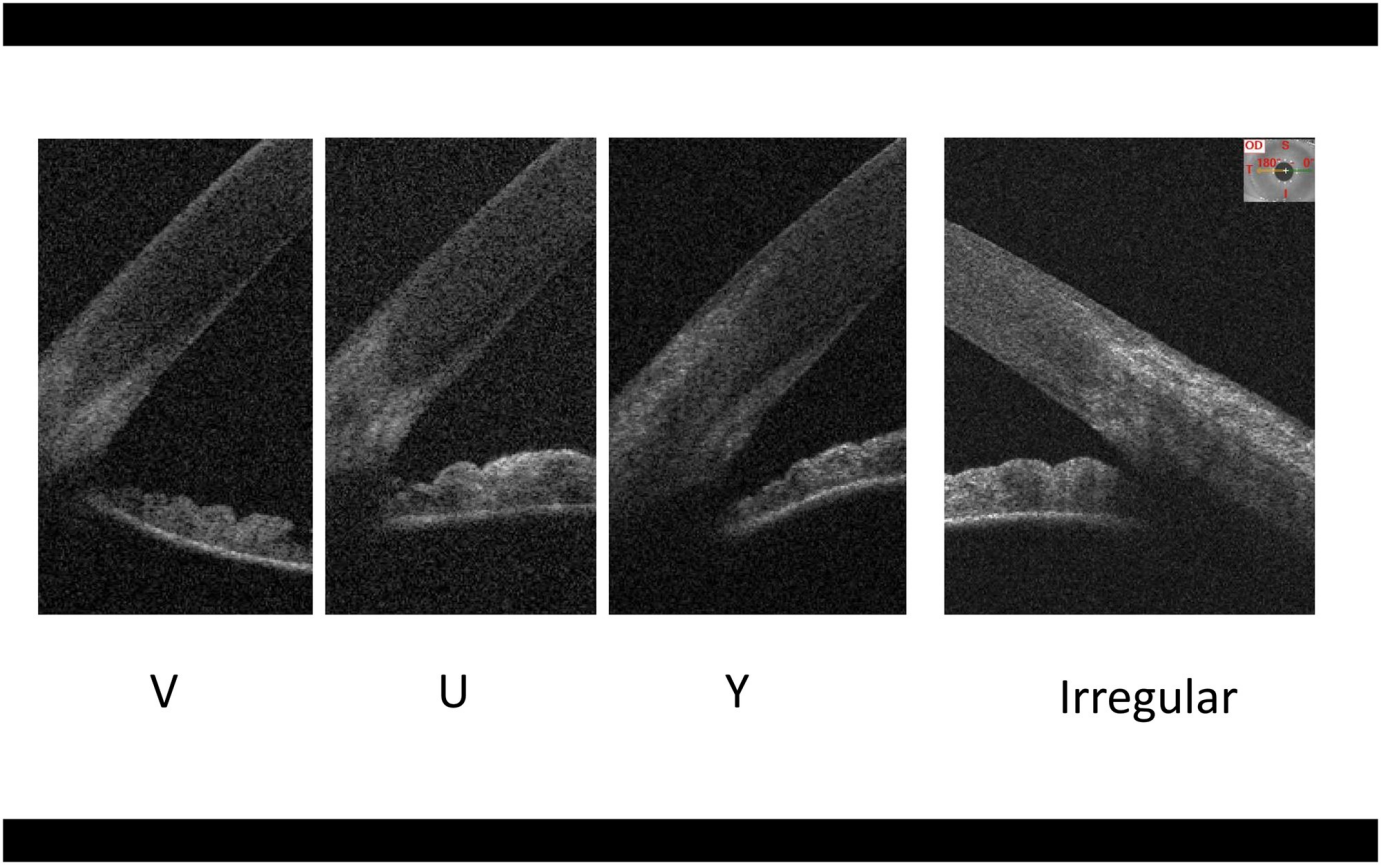
The shape of corneo-scleral junction. Examples of different tomographic reflectivity patterns of corneo-scleral junction at the horizontal corneal meridian: V-, U- and Y-shaped transition (temporal side); irregular transition (nasal side).

The shape of CSJ as a form of corneal tissue “insertion” into scleral tissue was assessed and assigned to 3 following patterns: V, U, Y. When no visible pattern was seen, the shape was described as irregular (Fig 2).

### Statistics

For each patient, only the right eye was analysed to offset any interocular dependency issues and statistical bias due to enantiomorphism [5]. According to other studies, ocular anterior segment parameters are mirrored [6, 7]. For parameters with both nasal and temporal measures, both sides were analysed separately.

Descriptive statistics, such as mean, standard deviation, median, minimum, maximum, interquartile range and coefficient of variation, were calculated for demographic and biometric measures. The normal distribution of continuous data was examined using the Shapiro–Wilk’s W test, using a critical value of 0.05. Since most of the variables showed significant variation from the normal distribution, Spearman’s rank-order correlation coefficient (Spearman’s rho) was used to assess the relationship between them. Univariate and multivariate linear regression analyses of anterior segment parameters with age and refractive error were performed (independent variables: age and refractive status; dependent variables: measured anterior segment parameters). The Wilcoxon signed-rank test was used to compare CSJt with CSJn. The nonparametric ANOVA and post hoc Kruskal-Wallis rank sum test was used for multiple pairwise comparisons between groups of eyes with the same CSJ pattern. To assess inter- and intraobserver repeatability and reproducibility of CSJt and CSJn manual measurements the intraclass correlation coefficient (ICC) was calculated. A value of 1 would represent perfect agreement, and a value of 0 would represent no agreement. Bland-Altman mean difference and 95% confidence interval (CI) were evaluated. Statistical analyses were performed using the software package Statistics 13 (StatSoft Poland).

## Results

### Descriptive statistics

101 right eyes of 101 patients (63 women, 38 men) were analysed. The median age was 33 years (range 18–55). The median spherical equivalent of cycloplegic refraction was -2.5 D (range -17.0 D to +7.5 D). Descriptive statistics of analysed parameters are presented in Table 1.

**Table 1:** Descriptive statistics of analysed ocular anterior segment variables: Mean, standard deviation (SD), median, minimum (Min), maximum (Max), interquartile range (IR) and coefficient of variation (CV).

Variable		Mean	SD	Median	Min	Max	IR	CV %
CSJt	mm	1.61	0.24	1.62	1.13	2.22	0.32	15.0
CSJn		1.19	0.18	1.18	0.73	1.80	0.24	14.9
CSJ Δ		0.43	0.22	0.42	0.03	1.10	0.35	51.7
HVID		11.74	0.37	11.81	10.84	12.83	0.51	3.2
SS-SS		12.18	0.41	12.19	11.22	13.24	0.50	3.4
CCT		0.550	0.035	0.547	0.494	0.644	0.044	6.2
CTt		0.610	0.039	0.608	0.536	0.728	0.051	6.3
CTn		0.667	0.044	0.658	0.598	0.793	0.068	6.6
AD		3.03	0.35	3.07	2.18	3.94	0.46	11.7
K1 ant		7.91	0.27	7.90	7.41	8.62	0.36	3.4
K2 ant		7.64	0.3	7.65	6.93	8.92	0.37	3.9
K1 post		6.68	0.23	6.70	6.29	7.2	0.36	3.5
K2 post		6.26	0.28	6.28	5.59	7.48	0.37	4.5
AOD 500 t		0.54	0.19	0.52	0.19	1.11	0.24	35.2
AOD 500 n		0.55	0.20	0.54	0.15	1.05	0.27	36.6
AOD 750 t		0.73	0.26	0.71	0.21	1.51	0.36	36.8
AOD 750 n		0.54	0.25	0.54	0.17	1.28	0.35	35.0
TISA 500 t	mm^2^	0.21	0.09	0.19	0.07	0.47	0.10	41.5
TISA 500 n		0.22	0.08	0.21	0.05	0.42	0.11	39.1
TISA 750 t		0.38	0.16	0.35	0.11	0.91	0.19	41.2
TISA 750 n		0.39	0.14	0.37	0.09	0.71	0.19	37.0

AD = aqueous depth; AOD = angle opening distance; CCT = central corneal thickness; CT = peripheral corneal thickness; CSJ = corneo-scleral junction; CSJΔ = CSJt-CSJn; HVID = horizontal visible iris diameter; K1 ant = flat anterior keratometry; K2 ant = steep anterior keratometry; K1 post = flat posterior keratometry; K2 post = steep posterior keratometry; SS-SS = scleral spur to scleral spur distance; TISA = trabecular space area; t = temporal; n = nasal.

### CSJ width

**Fig 3:**
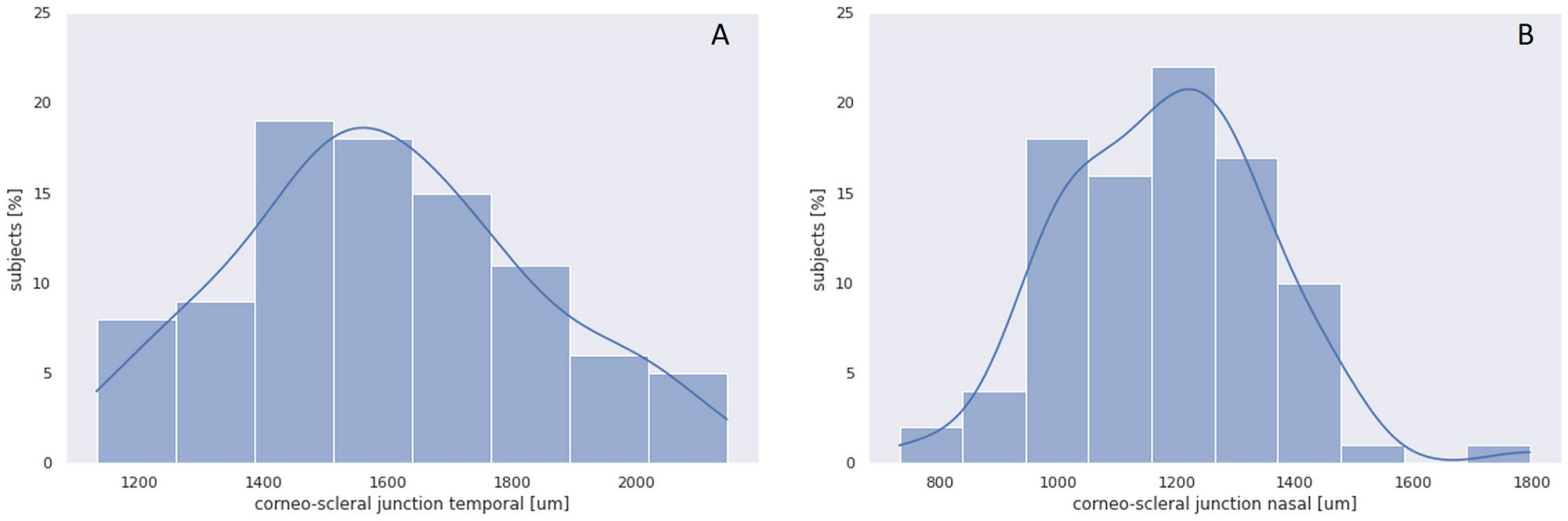
A, B. Frequency distribution of the corneo-scleral junction width. (A) temporal (B) nasal.

**Fig 4:**
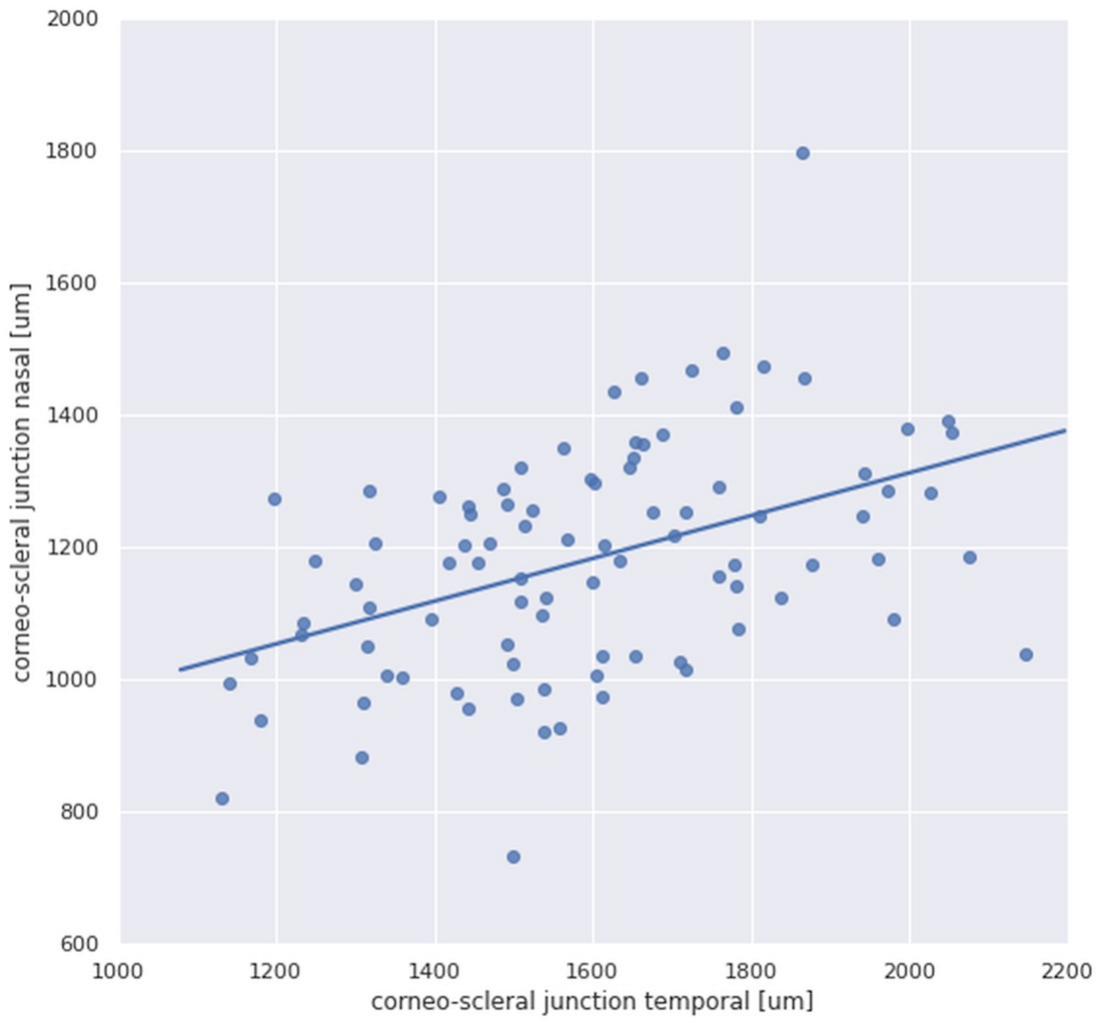
Correlation between corneo-scleral junction temporal width and nasal width (Spearman R = 0.46, *p*<.00001).

The CSJ was wider at temporal side (median 1.62 mm; range 1.13–2.22) than at nasal side (median 1.18 mm; range 0.73–1.80 mm) (*p*<.00001, Wilcoxon signed-rank test), with a median difference of 0.42 mm (range 0.03–1.1). The frequency distribution of CSJt and CSJn are presented on Fig 3A and 3B. The variability of the CSJ width was comparable on both sides (15%). CSJt width was positively correlated with CSJn width (R = 0.46, *p*<.00001) (Fig 4). The width of CSJt correlated with CSJΔ (R = 0.67, *p*<.0000).

The CSJt width showed negative correlation with ipsilateral angle parameters (AOD and TISA). There was also a weak, statistically significant positive correlation between CSJt width and HVID (R = 0.25, *p* = 0.009). These relationships were not statistically significant for CSJn. Correlations between both nasal and temporal CSJ with AOD, TISA, HVID, SS-SS and K1 ant are summarised in Table 2. Other parameters showed no significant relationships with CSJ width.

**Table 2:** CSJt and CSJn correlations with corresponding angle parameters (AOD, TISA), HVID, SS-SS and K1 ant.

Correlation		R (Spearman)	*P*
CSJt	AOD 500 t	-0.24	0.014*
	AOD 750 t	-0.22	0.021*
	TISA 500 t	-0.29	0.003*
	TISA 750 t	-0.28	0.009*
	HVID	0.25	0.009*
	SS-SS	0.09	0.34
	K1 ant	0.19	0.05
CSJn	AOD 500 n	-0.18	0.07
	AOD 750 n	-0.16	0.11
	TISA 500 n	-0.16	0.16
	TISA 750 n	-0.16	0.21
	HVID	0.05	0.56
	SS-SS	0.00	0.92
	K1 ant	0.19	0.04*

AOD = angle opening distance; CSJ = corneo-scleral junction; HVID = horizontal visible iris diameter; K1 ant = flat anterior meridian; TISA = trabecular space area; SS-SS = scleral spur to scleral spur distance; t = temporal; n = nasal.

Statistically significant *P* values (< .05) are marked with asterisk (*).

HVID was strongly correlated with SS-SS distance (R = 0.8; *p*< 0.000). The median difference between SS-SS and HVID was 0.45 mm (range 0.0–1.0 mm). Statistically significant correlations between SS, HVID and other parameters are summarised in Table 3.

**Table 3:** Statistically significant correlations of HVID and SS-SS with other anterior segment parameters.

Correlation	R (Spearman)	*P*
HVID & CTt	-0.20	.042
HVID & CTn	-0.21	.031
HVID & AD	0.45	< .001
HVID & K1 ant	0.43	< .001
HVID & K2 ant	0.39	< .001
SS-SS & HVID	0.80	< .001
SS-SS & K1 ant	0.41	< .001
SS-SS & K2 ant	0.46	< .001
SS-SS & K1 post	0.59	< .001
SS-SS & K2 post	0.55	< .001
SS-SS & CTt	-0.27	< .001
SS-SS & CTn	-0.30	< .001
SS-SS & AD	0.66	< .001
SS-SS & AOD 500 t	0.31	< .001
SS-SS & AOD750 t	0.29	< .001
SS-SS & AOD 500 n	0.29	< .001
SS-SS & AOD750 n	0.39	< .001

AD = aqueous depth; AOD = angle opening distance; CT = peripheral corneal thickness; HVID = horizontal visible iris diameter; K1 ant = flat anterior keratometry; K2 ant = steep anterior keratometry; K1 post = flat posterior keratometry; K2 post = steep posterior keratometry; SS-SS = scleral spur to scleral spur distance; TISA = trabecular space area; t = temporal; n = nasal.

The multivariate linear regression analyses of anterior segment parameters with age and refractive error confirmed the independent and significantly negative relationship between AD, TISA and AOD parameters with more hyperopic refractive status and older age (R = 0.31–0.39, *p*<.001) (Table 4). CSJt, CSJn, SS-SS, HVID, CCT, CTt, CTn showed no significant correlations with age or refractive error.

**Table 4:** Multivariable linear regression analysis of the relationship between age and spherical equivalent refractive error to aqueous depth, angle opening distance and trabecular space area at temporal and nasal side of horizontal AS-OCT scan.

Dependent variable	Side	R^2^	*P*	Independent variable	Non-standardised Beta (95% CI)	Standardised Beta	*P*
AD	n/a	0.37	<0.001	Age	-0.41 (-0.56;0.24)	0.08	< .001
				SE	-0.42 (-0.58;0.26)	0.08	< .001
AOD 500	Temporal	0.33		Age	-0.28 (-0.44;-0.11)	0.08	0.001
				SE	-0.47 (-0.64;-0.31)	0.08	< .001
	Nasal	0.33		Age	-0.31 (-0.48;-0.14)	0.08	< .001
				SE	-0.46 (-0.63;-0.29)	0.08	< .001
TISA 500	Temporal	0.31		Age	-0.26 (-0.43;-0.10)	0.08	0.002
				SE	-0.47 (-0.64;-0.30)	0.08	< .001
	Nasal	0.31		Age	-0.34 (-0.51;-0.17)	0.08	< .001
				SE	-0.40 (-0.58;-0.23)	0.08	< .001
AOD 750	Temporal	0.39		Age	-0.29 (-0.45;-0.13)	0.08	< .001
				SE	-0.53 (-0.69;-0.37)	0.08	< .001
	Nasal	0.35		Age	-0.32 (-0.49;-0.16)	0.08	< .001
				SE	-0.47 (-0.63;-0.30)	0.08	< .001
TISA 750	Temporal	0.38		Age	-0.28 (-0.44;-0.12)	0.08	< .001
				SE	-0.52 (-0.68;-0.36)	0.08	< .001
	Nasal	0.34		Age	-0.36 (-0.53;-0.20)	0.08	< .001
				SE	-0.41 (-0.58;-0.25)	0.08	< .001

AD = aqueous depth; AOD = angle opening distance; SE = spherical equivalent refractive error; TISA = trabecular space area.

Finding and determining the position of 2 lines separating the CSJ zone from the transparent cornea and the non-transparent sclera was easier at the temporal than at the nasal side of the scan: (1) easy determination—72% versus 47%; (2) moderate ease—20% versus 31%; (3) difficult to determine– 8% versus 22%, respectively. Irregular CSJ shape was the main cause of difficulty in determination.

The mean ICC for intraobserver reproducibility of CSJt and CSJn measurements was 0.99 (mean difference 0.069 mm and 0.068 mm, respectively) for investigator 1 (MMW). The interobserver agreement was high, with mean difference of -0.002 mm (95% CI 0.96) for CSJt and 0.018 mm (95% CI 0.95) for CSJn (Table 5).

**Table 5:** Inter- and intra-observer agreement of manual CSJt and CSJn measurements.

Agreement	Measurement	Bland-Altman analysis		ICC
		mean difference (mm)	95% CI limits* (mm)	95% CI
Inter-observer	CSJt	-0.002	-0.175, 0.179	0.96 (0.91, 0.98)
	CSJn	0.018	-0.099, 0.134	0.95 (0.88, 0.98)
Intra-observer 1	CSJt	-0.069	-0.141, 0.002	0.99 (0.98, 1.00)
	CSJn	-0.068	-0.121, -0.015	0.99 (0.98, 1.00)
Intra-observer 2	CSJt	-0.076	-0.145, -0.008	0.98 (0.95, 0.99)
	CSJn	-0.083	-0.144, -0.022	0.98 (0.96, 0.99)

ICC–intraclass correlation coefficient.

Observer 1 = MMW; Observer 2 = SW.

*Observer 2—Observer 1.

### CSJ shape

The pattern of CSJt was V-shaped in 56%, U-shaped in 15%, Y-shaped in 11% and irregular in 18% of cases. These proportions at the nasal side were as follows: V-shape 23%, U-shape 27%, Y-shape—3%, irregular—47% of cases. Generally, CSJn was shorter and more irregular than CSJt.

**Fig 5:**
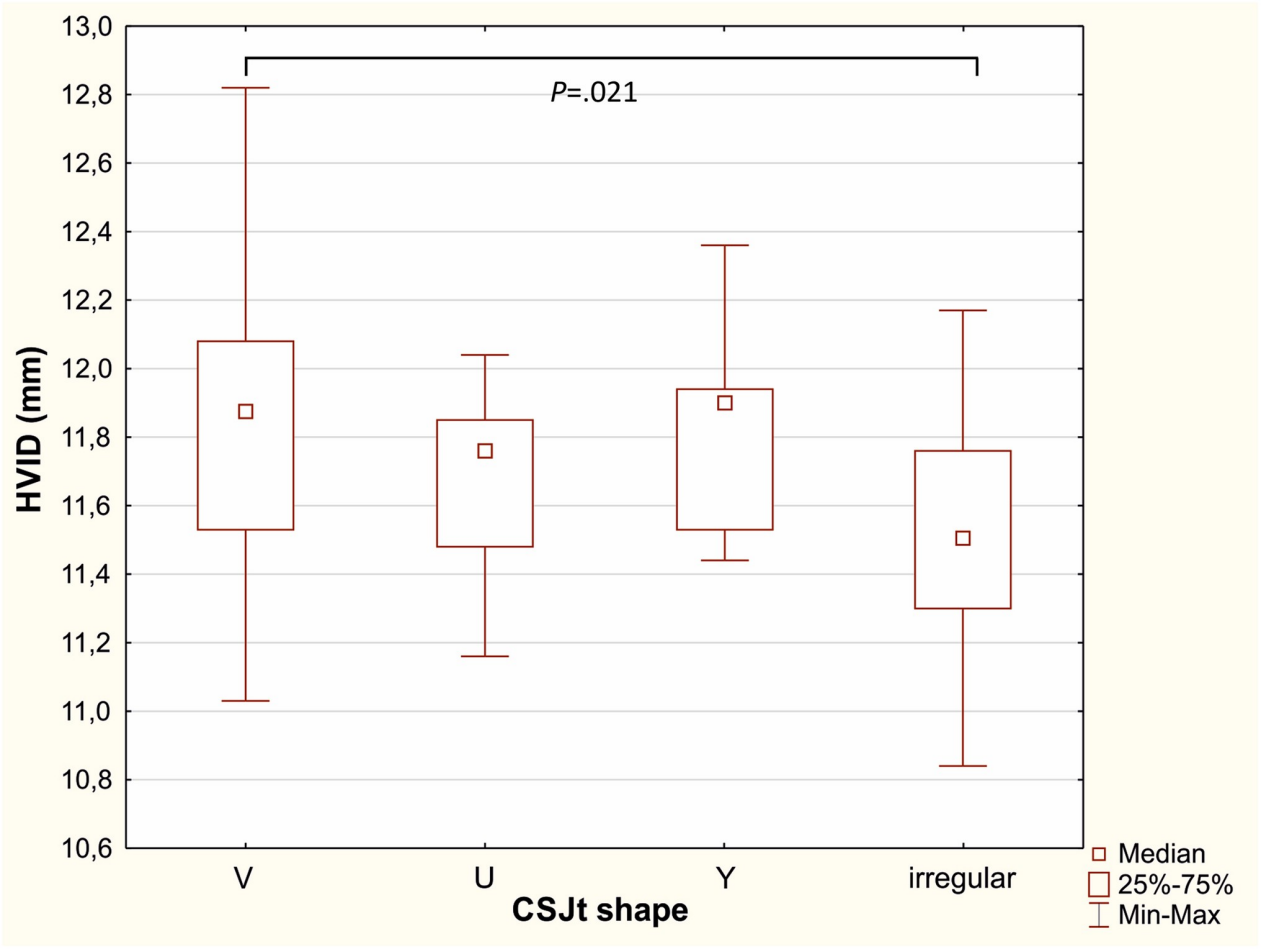
Box-plots of horizontal visible iris diameter (HVID) in eyes with different temporal corneo-scleral junction shape: V, U, Y and irregular (*P* = .021, Kruskal-Wallis test).

The eyes with V-shaped CSJt had significantly larger HVID (median 11.88 mm; 11.03–12.82 mm), than the eyes with irregular CSJt (median 11.50 mm; 10.84–12.17) (*p* = .021, Kruskal-Wallis test) (Fig 5). Other differences between eyes with different CSJ shape did not reach a statistical significance.

## Discussion

The greyscale limbus detection, based on difference between white sclera and darker iris image, is influenced by corneal transparency. Our study shows that the limbal transition zone, here called the corneo-scleral junction, is wider temporally than nasally and that there is a significant variability in the dimensions and cross-sectional reflectivity pattern of this area, visible at the AS-OCT horizontal scans. Our data imply that the greyscale method may not be the best way of determining the border between cornea and sclera.

The automated WTW measurements offer higher precision than manual methods, although the results from different devices vary significantly [1–8]. Modern optical biometers, such as IOLMaster 700 (Zeiss), Lenstar 900 (Haag-Streit), Argos (Alcon) or Heidelberg Anterion (Heidelberg Engineering), use high resolution photography and a reference image of the eye for the WTW assessment. The limits of agreement between these devices exceed 1.0 mm and the repeatability may be as low as 0.65 mm [2, 9–11].

The rate of change in transparency differs from eye to eye and the loss of transparency is not uniform across the depth of the cornea [6]. En-face imaging additionally complicates the measurements of the CSJ, which is an angled structure. For the WTW, the chord value between certain points must be calculated. These points are located within the transition between clear cornea and opaque sclera. The transition zone is not equally wide along the corneal circumference. Newer research using a corneo-scleral topographer revealed that the limbal sclera is rotationally asymmetric and variable between subjects [12]. Our data, obtained from AS-OCT scans, support these findings.

Optical tomography scans offer more exactitude than reference image, although the length of light used in OCT influences the penetration through tissues and visibility of the iris. AS-OCT using longer wavelength of 1310 nm penetrates well through sclera and reveals many details of the iridocorneal angle, but the determination of CSJ features may be compromised due to the relatively low resolution. The spectral-domain OCT using a wavelength of 845 nm is suboptimal for imaging the angle due to limited penetration through scattering tissue such as the sclera. However, this feature facilitates determination of the exact scleral tissue border at the limbus, by forming optical shadowing. In our study this shadowing helped to find the borders of CSJ with irregular transition pattern, which was present in 47% of cases at the nasal side and in 18% of cases at the temporal side.

The limbal transitional region contains two types of tissues with different light scattering properties. Multiple scattering in turbid media produces image noise, challenging the accuracy of both the automated and manual measurements. In our cohort, the temporal CSJ, wider and clearly V or U-shaped in most of the cases, was easier to evaluate, in opposition to the irregular pattern of the nasal side (difficult determination in 22% of cases). We also found, that eyes with regular reflectivity pattern had a larger HVID in comparison to eyes with irregular CSJ reflectivity pattern. This may suggest lower accuracy of HVID measurements in eyes with smaller corneas. Further studies would be necessary to evaluate these findings.

The CSJt width showed significant negative correlation with ipsilateral angle parameters (AOD 500, AOD 750, TISA 500, TISA 750), which may reflect the anatomical pattern of this area—the narrower the anterior chamber angle, the longer the transition between cornea and sclera. Such correlation was not seen at the nasal side. Interestingly, other authors, who assessed external corneo-scleral angle (profile) also found no significant correlation on the nasal side with anterior segment parameters, although on the temporal side correlation was noticed for WTW, anterior chamber depth and volume [13]. It is unclear, why the temporal side showed some relationship with other variables and the nasal side did not. There were some suggestions that horizontal asymmetry in the corneo-scleral transition might be related to the insertion of the medial rectus muscle [14]. It seems that the anterior segment architecture is generally slightly asymmetric, including cornea, sclera and crystalline lens tilt [12]. We found that CSJ on the nasal side was shorter, more difficult to assess and presented mostly an irregular pattern.

The CSJ width was poorly correlated with other known anterior segment parameters, with only weak positive correlation between CSJt and HVID (R = 0.25, *p* = 0.009). Segui-Crespo et al. showed a similar correlation between outer corneo-scleral angle and WTW, although they looked at the junction external profile only [13].

The HVID/WTW value for intraocular lenses calculations could be potentially replaced by more accurately measured angle to angle (ATA) or SS-SS distance. We found a strong, significantly positive correlation between HVID and SS-SS (R = 0.8, *p*<.001) in our group. Additionally SS-SS was correlated with other anterior segment parameters such as anterior and posterior K (R = 0.41–0.59, *p*<.001) and AD (R = 0.66, *p*<.001). Some weaker correlations were also observed between SS-SS and CCT and angle parameters while HVID correlations with other parameters were weaker or not significant (Table 3). For phakic implantable collamer lens (ICL) sizing, new calculation formulas have been developed, utilizing SS-SS or ATA distance and rejecting WTW value due to its lower reproducibility [15–17]. Nevertheless, the original algorithm, recommended by the ICL manufacturer (STAAR Surgical AG), is still based on WTW distance.

With regard to age and refractive error, no significant correlations with CSJ, as well as with HVID, SS-SS or CCT was observed. A study performed on a new swept-source AS-OCT (ANTERION, Heidelberg Engineering) similarly reported non-significant relationship between age, SE, CCT and SS-SS [18]. However, our data show an independent and significant negative relationship between AD, AOD, TISA and more hyperopic refractive status and older age, as expected.

The CSJ is often illustrated as a corneo-scleral profile, especially in contact lens fitting research [13]. Jesus at al. described the topographic method, using Eye Surface Profiler (ESP, Eaglet-Eye), showing the discrepancies between image-only and 2-D or 3-D techniques [19]. Our data show that the CSJ width has no significant correlation with the anterior corneal radius measured for a large, 7 mm zone; therefore the topographic method seems to be less useful for the estimation of limbus architecture than the AS-OCT technique.

The main limitation of this study was the subjective method of determining the width of CSJ at the AS-OCT horizontal scan. Nevertherless, the repeatiblity and reproducibility of solely manual CSJ measurements were high. The spectral domain AS-OCT high resolution, image magnification and optical shadowing of iris from the non-transparent scleral tissue helped to increase accuracy (Fig 1).

The AS-OCT findings resemble the histologic limbus cross-section. The junction follows a curvilinear diagonal from anterior to posterior and then abruptly curves anteriorly up to the Schlemm’s canal [20]. The advantage of latest OCT technology is the precise in vivo visualization of the three-dimensional transparency profile of this area. This should help to enhance the accuracy of WTW measurements. Surprisingly, all OCT based ophthalmic devices still use the en-face image. We are convinced, that there is a lot of space for improvement.

## Conclusions

In conclusion, the CSJ transverse section analysis may help to understand the variability of the en-face measurements of WTW distance (limbus to limbus), which is an important number used in intraocular lens calculation formulas and for posterior chamber phakic lens sizing. There is a significant horizontal asymmetry of corneo-scleral junction width and shape. The image based, en-face WTW assessments could be replaced by OCT-based measurements.

## Supporting information

S1 File Data. (XLSX)
